# *Mycobacterium bohemicum* and Cervical Lymphadenitis in Children

**DOI:** 10.3201/eid1407.080142

**Published:** 2008-07

**Authors:** Julia Huber, Elvira Richter, Lothar Binder, Matthias Maa, Robert Eberl, Werner Zenz

**Affiliations:** * Medical University of Graz, Graz, Austria; † National Reference Center for Mycobacteria, Borstel, Germany; ‡ Elisabethinen Hospital, Linz, Austria; § University Hospital Salzburg, Salzburg, Austria

**Keywords:** Mycobacterium bohemicum, nontuberculous mycobacterium, cervical lymphadenitis, letter

**To the Editor:** Members of the genus *Mycobacterium* are well-established causes of granulomatous lymphadenitis in children. *M. bohemicum* was first described in 1998 in a patient with Down syndrome (*1*). The organism is characterized by a unique 16S rRNA gene sequence (*1*) and has been isolated from humans, animals, and the environment (*2*). Published data on *M. bohemicum* are limited to the original species description and 5 additional case reports (*3*–*6*).

We report on 4 cases of cervical lymphadenitis caused by *M. bohemicum* that occurred in 4 children (2 boys, 2 girls) from Austria during 2002–2006. Age range of the children was 2.5–3.5 years (Table). Each child was admitted to the hospital with a history of 2–3 weeks of submandibular swelling that did not respond to oral antimicrobial therapy.

**Table:** Characteristics of 4 children with cervical lymphadenitis caused by *Mycobacterium bohemicum,* Austria, 2002–2006*

Patient no.	Sex; age	Clinical findings	Therapy	Histologic findings
1	M; 2 y, 10 mo	Right submandibular swelling	Incomplete surgical excision; clarithromycin and rifabutin for 3 mo	Granulomatous and partly necrotizing inflammation, multiple giant cells and perinodal fibrosis; no acid-fast bacilli
2†	F; 2 y, 9 mo	Right submandibular swelling	Total lymph node excision	Granulomatous and partly necrotizing inflammation; acid-fast bacilli
3	F; 3 y	Angular right-sided swelling	Total lymph node excision	Granulomatous and partly necrotizing inflammation; sporadic findings of acid-fast bacilli
4	M; 3 y, 7 mo	Swelling in right mandibular area	Total lymph node excision	Granulomatous and partly necrotizing inflammation; no acid-fast bacilli

*All patients had negative PCR results for *Mycobacterium tuberculosis* complex; all patients recovered completely with no relapse. †Patient had history of psychomotor disorder after perinatal asphyxia.

Physical examination showed enlarged cervical lymph nodes under normal skin. Laboratory evaluation showed leukocyte and differential counts within normal limits and a negative result (<2 mg/L) for C-reactive protein. Ultrasonography demonstrated enlarged lymph nodes with heterogeneous echo and a central lacuna-like lesion.

Lymph nodes were excised from each patient. Histologic examination of the nodes demonstrated granulomatous and partly necrotizing inflammation (Table). PCR results for *M. tuberculosis* complex were negative. For 3 patients, the affected lymph nodes were completely removed and no antimicrobial therapy was prescribed. For the other patient, removal of the affected lymph node was incomplete, and clarithromycin and rifampicin for 3 months were prescribed**.** All patients remained healthy for >12 months after therapy.

From all patients, parts of the excised lymph nodes were directly used for acid-fast smear and culture for mycobacteria. All specimens were processed according to national guidelines (the German Institute for Standardization [DIN], Diagnostics for Tuberculosis DIN 58943-3, Cultural Methods for Isolation of Mycobacteria, Berlin: Beuth; 1996.)

Slides for microscopic examination were prepared directly from minced lymph nodes before decontamination, and the tissue was stained according to the Ziehl-Neelsen technique. Each sample was decontaminated, homogenized, and concentrated by using the N-acetyl-L-cysteine-sodium hydroxide method. Samples were spread onto solid slants (Löwenstein-Jensen, Stonebrink; National Reference Center for Mycobacteria, Borstel, Germany) and inoculated into BACTEC Mycobacteria growth indicator tubes (MGIT 960; Becton Dickinson and Company, Cockeysville, MD, USA). Solid slants were incubated at 31°C and 37°C for 8 weeks and inspected for growth weekly. MGITs were incubated in and automatically read with a BACTEC MGIT instrument.

Results of nucleic acid amplification for *M. tuberculosis* complex by using the BD ProbeTec system (Becton Dickinson and Company) as well as the amplification of DNA coding for the mycobacterial 16S rRNA gene were negative. Growth of mycobacteria was detected after 12–17 days in liquid MGIT cultures. For species identification of the cultures, part of the 16S rRNA gene was sequenced (*7*). The resulting sequences were compared with those in international databases (www.ridom.de; www.ncbi.nlm.nih.gov/BLAST). The strains were identified as *M. bohemicum*.

To date, >125 nontuberculous mycobacteria species have been described (www.bacterio.cict.fr/m/mycobacterium.html). Nontuberculous mycobacterial cervical lymphadenitis is most frequently caused by *M. avium* (80%), *M. malmoense*, *M. kansasii, M. lentiflavum*, *M. haemophilum,* and *M. scrofulaceum* (*8*). Because the phenotypic characteristics of *M. bohemicum* closely resemble those of *M. scrofulaceum*, these species can easily be misidentified by analysis of biochemical and cultural features only. The technique by which *M. bohemicum* can clearly be identified is sequence analysis (*8*,*9*).

Other than in the 4 patients reported here, *M. bohemicum* infection has only been reported in 5 patients worldwide (*3*–*6*). Each was a child with laterocervical and submandibular lymphadenitis. Total lymph node excision was performed with a good outcome for all patients except 1, who required additional treatment with antimicrobial drugs because the infected lymph node was incompletely excised (*4*). Additionally, a systemic *M. bohemicum* infection associated with immunodeficiency was reported recently (*10*). Treatment recommendations for nontuberculous mycobacterial lymphadenitis are outlined in discussions of individual nontuberculous mycobacterium species. Guidelines for localized lymphadenitis caused by any nontuberculous mycobacterium species recommend complete surgical excision of the involved lymph nodes (*8*). Additional antimicrobial drug therapy is recommended only for patients for whom removal was incomplete (*8*). Our patient who received combination antimicrobial drug treatment improved, with no relapse.

In summary we report 4 cases of *M. bohemicum* from Austria, a country with 8 million inhabitants. Because these cases were observed in a relatively small country, infections with *M. bohemicum* may be more common than previously thought. More such cases may be discovered as a result of improved microbiologic diagnostic techniques. We believe that *M. bohemicum* should be listed among the species that induce nontuberculous mycobacterial infections.
